# B-type natriuretic peptide and high sensitive C-reactive protein predict 2-year all cause mortality in chest pain patients: a prospective observational study from Salta, Argentina

**DOI:** 10.1186/1471-2261-11-57

**Published:** 2011-09-29

**Authors:** Ricardo León de la Fuente, Patrycja A Naesgaard, Stein Tore Nilsen, Leik Woie, Torbjoern Aarsland, Patricio Gallo, Heidi Grundt, Harry Staines, Dennis WT Nilsen

**Affiliations:** 1 Cardiology Research Institute, Catholic University of Salta, Salta, Argentina; 2 Department of Cardiology, Stavanger University Hospital, Stavanger, Norway; 3 Department of Research, Stavanger University Hospital, Stavanger, Norway; 4 Institute of Clinical Medicine, University of Bergen, Bergen, Norway; 5 Department of Medicine, Stavanger University Hospital, Stavanger, Norway; 6 Sigma Statistical Services, Balmullo, UK; 7 Institute of Medicine, University of Bergen, Bergen, Norway

## Abstract

**Background:**

Several mechanisms are involved in the pathophysiology of the Acute Coronary Syndrome (ACS). We have addressed whether B-type natriuretic peptide (BNP) and high-sensitive C-reactive protein (hsCRP) in admission samples may improve risk stratification in chest pain patients with suspected ACS.

**Methods:**

We included 982 patients consecutively admitted with chest pain and suspected ACS at nine hospitals in Salta, Northern Argentina. Total and cardiac mortality were recorded during a 2-year follow up period. Patients were divided into quartiles according to BNP and hsCRP levels, respectively, and inter quartile differences in mortality were statistically evaluated applying univariate and multivariate analyses.

**Results:**

119 patients died, and the BNP and hsCRP levels were significantly higher among these patients than in survivors. In a multivariable Cox regression model for total death and cardiac death in all patients, the hazard ratio (HR) in the highest quartile (Q4) as compared to the lowest quartile (Q1) of BNP was 2.32 (95% confidence interval (CI), 1.24-4.35), p = 0.009 and 3.34 (95% CI, 1.26-8.85), p = 0.015, respectively. In the TnT positive patients (TnT > 0.01 ng/mL), the HR for total death and cardiac death in Q4 as compared to Q1 was 2.12 (95% CI, 1.07-4.18), p = 0.031 and 3.42 (95% CI, 1.13-10.32), p = 0.029, respectively.

The HR for total death for hsCRP in Q4 as compared to Q1 was 1.97 (95% CI, 1.17-3.32), p = 0.011, but this biomarker did not predict cardiac death (p = 0.21). No prognostic impact of these two biomarkers was found in the TnT negative patients.

**Conclusion:**

BNP and hsCRP may act as clinically useful biomarkers when obtained at admission in a population with suspected ACS.

**Trial Registration:**

ClinicalTrials.gov Identifier: NCT01377402.

## Background

Myocardial injury in the ACS may be detected by an increase in the troponins, and among these TnT is considered to be a sensitive diagnostic marker in this condition. As the troponins are released as a result of myocardial necrosis secondary to thrombus formation in a coronary artery, they are not actively involved in the pathophysiology of ACS. Therefore, several patients with chest pain and ACS may exhibit normal levels of TnT in the subacute period preceding a myocardial infarction (MI).

The lack of diagnostic support during the early phases before the thrombus formation has stimulated the search for serum biomarkers, able to identify disease activity in patients with a negative TnT. Such markers may alert the clinician of an impending ACS in patients with chest pain without troponin release. Strategies to combine multiple biomarkers that may reflect diverse pathobiological contributors to the occurrence and complications of ACS have been an appealing approach to enhance risk assessment, targeting therapy more effectively. Proof-of-principle has been established with evidence for improved risk stratification using the combination of BNP, hsCRP and cardiac troponin [1-4].

BNP is a counter-regulatory peptide hormone predominantly synthesized in the ventricular myocardium, and is released into the circulation in response to ventricular dilatation and pressure overload [5,6]. It is a well known marker of left ventricular dysfunction and heart failure (HF) and it provides prognostic information beyond and above left ventricular ejection fraction (LVEF) as well as troponins in patients with ACS [7-10].

C-reactive protein (CRP) is an acute-phase reactant and a marker for underlying systemic inflammation, including atherosclerosis and plaque rupture with ensuing thrombus formation [11-13]. Through the use of appropriate high-sensitive assays, it has been possible to investigate the prognostic utility in cardiovascular disease (CVD) of plasma CRP levels previously considered to be within the normal range [14]. Burke and colleagues have suggested that hsCRP in serum reflects the numbers of vulnerable coronary atherosclerotic plaques in sudden cardiac death (SCD) [15]. Nevertheless, the prognostic value of this marker in CVD is still controversial [16,17] and its use in this setting should be restricted to subjects at high risk of CVD [1].

However, there are limited data available that prospectively compare these two biomarkers in admission samples from an unselected patient population presenting to the emergency department (ED) with chest pain. Furthermore, few trials have been conducted prospectively with this aim in an Argentinean population. In addition, their role in risk stratification of patients with ACS is still under evaluation, and therefore additional investigations are necessary.

The aims of this study were to explore the ability of BNP and hsCRP to predict risk of total and cardiac mortality within 24 months in a consecutively hospitalized patient population with chest pain and suspected ACS.

## Methods

### Study Design and Patient Population

This study was a regional multicenter prognostic study (ARRA-RACS: ARgentinean Risk Assessment Registry in the Acute Coronary Syndrome) designed to evaluate prospectively the prognostic impact of BNP and hsCRP in a population consisting of 982 patients with chest-pain and a suspected ACS, consecutively admitted to nine different hospitals in Salta, Argentina from December 2005 to January 2009. Eight centers were private and one was public. The latter included sixty-two patients (6.5%).

Patients entered into the registry were admitted for ACS as a presumptive diagnosis and had to be alive at the time of hospitalization. We used troponin-T levels at baseline and at six hours after admission for disease classification. Furthermore, BNP and hsCRP were determined in all patients as quality indicators in our registry.

Clinical data were collected at each site by a trained coordinator using a standardized nine-page case report form. Demographic characteristics, medical history, presenting symptoms, biochemical and electrocardiographic findings, treatment practices, and a variety of hospital outcome data were collected. Recorded information also included patient management data and outcome during hospitalization as well as after discharge. The patients received standard medical treatment at all centers. However, some patients needed to be referred to other more specialized hospitals with cardiac catheterization laboratory facilities when intervention was required.

Main exclusion criteria were age < 18 years, unwillingness or incapacity to provide informed consent and prior inclusion in the present study. Four patients with missing data for hsCRP were excluded from the present analysis.

The primary outcome measure of the present study was all-cause mortality from the time of inclusion until two year follow-up. The secondary outcome was cardiac death.

The term ACS in the present study encompasses unstable angina pectoris (UAP), Non ST-segment Elevation Myocardial Infarction (NSTEMI) and ST-segment Elevation Myocardial Infarction (STEMI). The following classification for the index diagnosis was used: STEMI; ST-segment elevation combined with TnT values > 0.03 ng/mL. NSTEMI; Transient ST-segment elevation, ST-segment depression, or T-wave inversion in at least 2 contiguous leads combined with TnT values > 0.03 ng/mL. UAP; Transient ST-segment depression or T-wave inversion and TnT values ≤ 0.03 ng/mL, or borderline TnT values above 0.01 ng/mL up to 0.03 ng/mL without ECG changes. No-ACS: All other conditions (i.e. unspecific chest pain, arrhythmias, atrial fibrillation etc.) without ECG changes and with negative troponins. The definition of cardiac death included death, preceded by a definitive myocardial infarction or by chest pain > 20 minutes without a given TnT, or a history of ischemic heart disease and no other obvious cause of death [18].

Survival status, date and cause of death were obtained by an office visit at 12 months and telephone interview at 30 days, 6, and 24 months during the 2-year follow-up period. The family, neighbours and the national registry department were contacted to obtain relevant information regarding relocations. Information related to cause of death was obtained through official records and additional information was supplied by close family members.

Laboratory parameters and clinical parameters, including age, gender, assessment of previous MI, angina pectoris, previous revascularizations [percutaneous coronary intervention (PCI) or coronary artery bypass graft (CABG)], congestive heart failure (CHF) according to Killip-Kimball class [19], diabetes mellitus (DM), smoking status (categorized as current smokers, previous smokers or never-smokers), hypercholesterolemia (defined as total cholesterol concentrations above 250 mg/dl or statin treated hypercholesterolemia), beta-blockers and arterial hypertension (defined as repeated blood pressure measurements above 140/90 mmHg or treated hypertension) were based on hospital records and personal interviews. Electrocardiographic (ECG) findings at admission were classified according to the presence of ST-segment changes (i.e. ST-segment depression or elevation, T-wave inversion or left bundle-branch block).

Written informed consent was obtained from all patients. The study was approved by the Ethics Committee at the Board of Medical School of Salta and conducted in accordance with the Helsinki declaration of 1971, as revised in 1983. At two hospitals the study also needed to be approved by a local hospital ethics committee or institutional review board. The Norwegian biobank containing Argentinean blood samples was approved by the Regional Board of Research Ethics and the Norwegian health authorities.

### Blood Sampling Procedures and Laboratory Measurements

Peripheral blood samples for determination of TnT, creatinine, glucose, lipids and hsCRP in serum and BNP in EDTA (ethylene diamine tetraacetic) acid plasma, were drawn immediately following admission by direct venipuncture of an antecubital vein, applying a minimum of stasis. A repeated blood sample for the determination of TnT was drawn six hours following the primary blood sample. Clotted whole blood and EDTA blood samples were centrifuged for 15 min with 2000 x g at 20°C without delay. Serum and EDTA plasma were immediately frozen in three aliquots, stored locally at -70°C and transferred in frozen condition (dry ice) to Stavanger, Norway in three different shipments after collection of the first 100 samples, the next 400 samples and finally the remaining samples, respectively. These samples remained stored in a Norwegian biobank at -70°C until measurements were performed.

TnT was quantified by a cardiac-specific second-generation troponin T ELISA assay from Roche diagnostics, using a high-affinity cardiac-specific TnT isoform antibody [20]. The lower detection limit of the assay used is 0.01 ng/mL. In this study a cut off level of 0.01 ng/mL was used with a coefficient of variation (CV) of 10%.

BNP was analysed in EDTA plasma using the Microparticle Enzyme Immunoassay (MEIA) Abbott AxSYM^® ^(Abbott Laboratories, Abbott Park, Illinois, USA). The dynamic range was 0-4000 pg/mL and the within-run coefficient of variation (CV) was 6.3% at 95 pg/mL and 4.7% at 1587 pg/mL, respectively.

HsCRP was measured with the use of an immunoturbidimetric assay (Tina-quant^® ^C-reactive protein (latex) high sensitive assay, Roche Diagnostics, Germany) performed on a Roche automated clinical chemistry analyzer (MODULAR P). The detection limit was 0.03 mg/L and the measuring range 0.1-20.0 mg/L with an extended measuring range with automatic re-run 0.1-300 mg/L. The between-assay CV was 3.45% at 1.19 mg/L and 2.70% at 0.43 mg/L, respectively.

### Statistical analysis

The patients were divided into quartiles according to their BNP and hsCRP levels. Approximately normally distributed variables were given as mean and standard deviation (SD), whereas variables with skewed distributions were given as median and quartiles. The Chi-square test for association was applied between both the BNP and hsCRP quartiles and categorical variables at baseline. The one-way ANOVA test was used to test for equality of means of scale variables (e.g. age) amongst quartiles, and the two-sample t test and Mann Whitney test were used for comparing the means and medians of two samples, respectively. The hazard ratios (HR) are presented with 95% confidence interval (CI). Stepwise Cox multivariable proportional hazards regression models with total death and cardiac death as the dependent variables and BNP, hsCRP quartiles and other variables as potential independent predictors (listed below) were fitted. To examine the differences in prognosis between subjects in the upper versus the lower quartile of BNP and hsCRP, we adjusted for age, sex, smoking, hypertension, index diagnosis, creatinine, diabetes mellitus, CHF (defined by Killip-Kimball class at admission, those patients in class 2 to 4 were classified as CHF patients and non CHF for class 1), history of previous CHD (i.e. history of either angina pectoris, MI, CABG or PCI), hypercholesterolemia/use of statins, TnT > 0.01 ng/mL and beta-blockers prior to enrolment. The Kaplan-Meier product limits were used for plotting the times to event. In the discriminate analyses BNP, CRP, log_e _(BNP) and log_e _(CRP) were used as individual variables. The statistical analyses were performed using the statistical package SPSS version 19.0. All tests were two-sided with a significance level of 5%.

## Results

A total of 982 patients were enrolled in the ARRA-RACS study. No hsCRP samples were available for four patients.

No patient was lost to follow-up at two years. At index hospitalization, 388 patients (39.5%) had a peak TnT concentration exceeding 0.01 ng/mL. 16.8% of the patients were in Killip-Kimball class 2-4 and six percent were in class 3 and 4.

The baseline characteristics of the patients, stratified according to BNP and hsCRP quartiles at admission are listed in Tables 1 and 2, respectively. The median BNP and hsCRP concentrations in plasma were 78.1 (35.8-179.7) pg/mL [25 and 75% percentiles] and 3.1 (1.3-8.4) mg/L [25 and 75% percentiles], respectively. Patients with BNP in the higher quartiles were significantly older and had a higher proportion of patients with a TnT exceeding 0.01 ng/ml. Furthermore, there were more past smokers and patients with established coronary heart disease and heart failure in the higher quartiles, and creatinine also increased significantly.

**Table 1:** Baseline characteristics for patient strata according to quartiles of BNP.

Quartiles of BNP pg/mL					
Characteristics	< 36	36-78	79-180	181-4000	p value
Age; years ± SD	57.9 ± 12.4	60.7 ± 11.5	62.7 ± 13.9	67.4 ± 13.8	0.000
Female n (%)	95(38.8)	96(39.0)	94(38.2)	109(44.5)	0.453
Hypertension n (%)	142(58.0)	157(63.8)	173(70.3)	162(66.1)	0.036
DM n (%)	46 (19.1)	45 (18.4)	43 (17.6)	68(28.3)	0.012
Current Smoker n (%)	90(37.5)	57(23.5)	51(20.9)	41(17.1)	0.000
Past Smoker n (%)	113(47.3)	130(53.9)	140(57.6)	154(64.4)	0.000
Angina Pectoris n (%)	45(18.4)	47(19.1)	68(27.6)	63(25.7)	0.028
CHF n (%) Killip-Kimball Class 2-4	33(13.5)	44(17.9)	33(13.4)	55(22.4)	0.021
MI n (%)	6(2.4)	21(8.5)	32(13)	35(14.3)	0.000
CABG n (%)	3(1.2)	10(4.1)	14(5.7)	20(8.3)	0.003
PCI n (%)	17(6.9)	23(9.3)	30(12.2)	28(11.4)	0.208
STEMI n (%)	36(14.9)	23(9.5)	40(16.5)	45(18.8)	0.030
TnT Positive n (%)	67(27.5)	65(26.4)	100(40.7)	156(63.7)	0.000
Creatinine n (%) (above median)	113(46.3)	111(45.1)	118(48.4)	143(58.6)	0.011
Cholesterol/Statin n (%)	31(12.7)	40(16.3)	49(19.9)	40(16.3)	0.191
Beta-blocker n (%)	53(21.9)	50(20.5)	76(31.1)	74(31.1)	0.006
CHD n (%)	56(23.0)	71(29.1)	98(40.0)	99(40.9)	0.000

BNP, brain natriuretic peptide; DM, diabetes mellitus; CHF, congestive heart failure; MI, myocardial infarction; CABG, coronary artery bypass grafting; PCI, percutaneous coronary intervention; STEMI, ST-elevation myocardial infarction; TnT, troponin-T; cholesterol = conc. > 250 mg/dl; CHD, coronary heart disease

**Table 2:** Baseline characteristics for patient strata according to quartiles of hsCRP.

Quartiles of hsCRP mg/L					
Characteristics	< 1.3	1.4-3.1	3.2-8.4	8.5-350.8	p value
Age; years ± SD	60.2 ± 13.7	60.9 ± 13.6	63.9 ± 12.2	63.7 ± 13.6	0.002
Female n (%)	85(34.7)	100(40.5)	113(46.3)	94(38.8)	0.070
Hypertension n (%)	160(65.3)	157(63.6)	162(66.4)	153(63.2)	0.870
DM n (%)	39(16)	45(18.6)	53(22.1)	64(26.7)	0.026
Current Smoker n (%)	58(24.2)	57(23.6)	67(27.9)	55(22.8)	0.572
Past Smoker n (%)	140(58.1)	139(58.2)	126(52.7)	130(54.4)	0.582
Angina Pectoris n (%)	48(19.6)	59(23.9)	60(24.6)	55(22.7)	0.563
CHF n (%) Killip-Kimball Class 2-4	29(11.8)	34(13.8)	51(20.9)	50(20.7)	0.009
MI n (%)	19(7.8)	22(8.9)	21(8.6)	31(12.8)	0.233
CABG n (%)	11(4.5)	12(5.0)	12(5.0)	11(4.6)	0.991
PCI n (%)	25(10.2)	29(11.7)	27(11.1)	16(6.6)	0.235
STEMI n (%)	24(10.0)	32(13.3)	45(18.9)	41(17.0)	0.029
TnT Positive n (%)	62(25.3)	82(33.3)	111(45.5)	130(53.7)	0.000
Creatinine n (%) (above median)	117(47.8)	115(46.6)	117(48.0)	136(56.2)	0.126
Cholesterol/Statin n (%)	43(17.6)	42(17.0)	42(17.2)	33(13.6)	0.621
Beta-blocker n (%)	78(32.1)	64(26.6)	57(23.8)	53(22.1)	0.064
CHD n (%)	71(29.1)	84(34.4)	86(35.70)	82(33.9)	0.435

hsCRP, high sensitive C-reactive protein; DM, diabetes mellitus; CHF, congestive heart failure; MI, myocardial infarction; CABG, coronary artery bypass grafting; PCI, percutaneous coronary intervention; STEMI, ST-elevation myocardial infarction; TnT, troponin-T; cholesterol = conc. > 250 mg/dl; CHD, coronary heart disease

The differences between the upper and lower quartiles of hsCRP were not quite as pronounced as for BNP. However, as for BNP, patients were older and a higher proportion of patients had TnT exceeding 0.01 ng/ml.

BNP, CRP, loge (BNP) and loge (CRP) were used as individual variables in a discriminate analysis with the purpose to identify patients with true ACS versus non-cardiac chest pain at index admission and to predict fatal outcome in the total population and in the TnT negative (TnT ≤ 0.01 ng/mL) and TnT positive (TnT > 0.01 ng/mL) subpopulations. In the univariate discriminate analyses, a TnT positive event at admission was correctly classified by BNP in 66.7% and by hsCRP in 64.2% of cases in their non-logarithmic form and slightly less in their logarithmic form. The specificity of non-logarithmic BNP and hsCRP for predicting all-cause mortality in the total population was 89.6% and 90.3%, respectively, with a sensitivity of 44.5% and 31.9%, respectively (Table 3).

**Table 3:** Discriminate analysis of all cause mortality using BNP, hsCRP and their natural logarithm, for total population, TnT positive patients, and TnT negative patients, respectively

Population		BNP	CRP	Log(BNP)	Log(CRP)
All	Specificity	89.6	90.3	71.2	69.6
	Sensitivity	44.5	31.9	65.5	61.0
	Overall	84.2	83.2	70.5	68.6
	p value	0.000	0.000	0.000	0.000
					
TnT positive	Specificity	86.5	85.7	65.3	65.8
	Sensitivity	41.5	32.9	67.1	56.8
	Overall	76.9	74.4	65.7	69.3
	p value	0.000	0.001	0.000	0.000
					
TnT negative	Specificity	88.1	92.2	69.1	65.5
	Sensitivity	40.5	24.3	61.8	51.4
	Overall	85.1	88.0	68.7	64.6
	p value	0.000	0.000	0.000	0.001

BNP, brain natriuretic peptide; hsCRP, high sensitive C-reactive protein; TnT, troponin-T

Combining the two predictors in our quartile comparisons did not increase the prognostic impact as compared to the separate analysis of BNP and hsCRP.

### All-Cause Mortality

#### Total Patient Population

**Figure 1:**
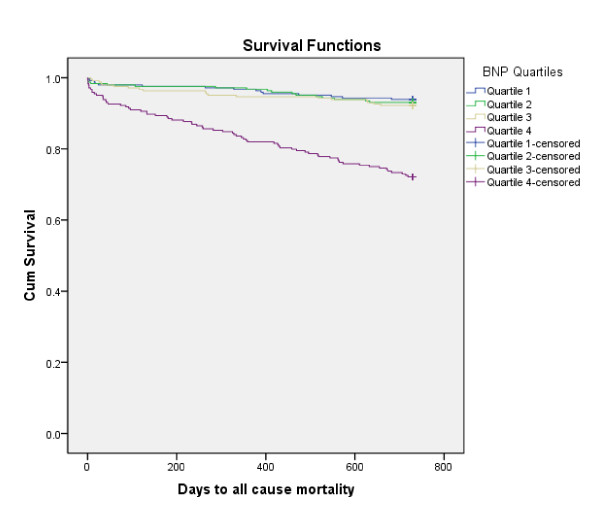
**Kaplan-Meier plots for the cumulative risk of total mortality for the BNP quartiles**.

**Figure 2:**
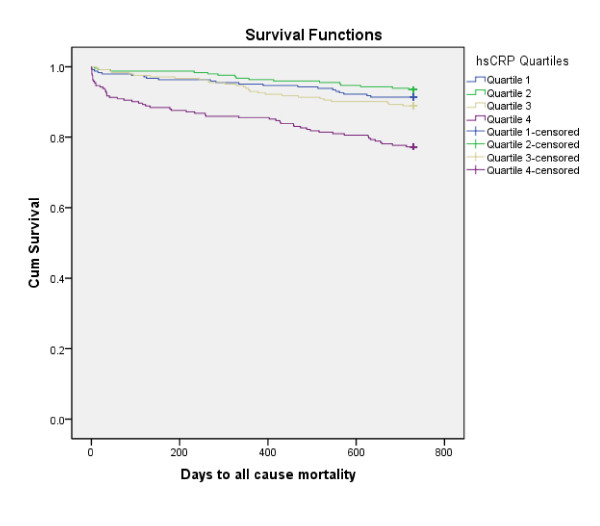
**Kaplan-Meier plots for the cumulative risk of total mortality for the hsCRP quartiles**.

After a follow-up period of 24 months, 119 patients (12.2%) had died. Kaplan-Meier survival curves for the primary endpoint according to BNP and hsCRP quartiles at baseline are presented in Figures 1 and 2, respectively, (the log rank test for each was 0.000). The BNP and hsCRP levels were significantly higher among patients dying than in 2-year survivors; 228 (66-603) versus 72 (34-148) pq/mL [median, 25 and 75% percentiles], p = 0.000 and 7.8 (2.3-35.6) versus 2.9 (1.3-7.5) mg/L [median, 25 and 75% percentiles], p = 0.000, respectively.

**Figure 3:**
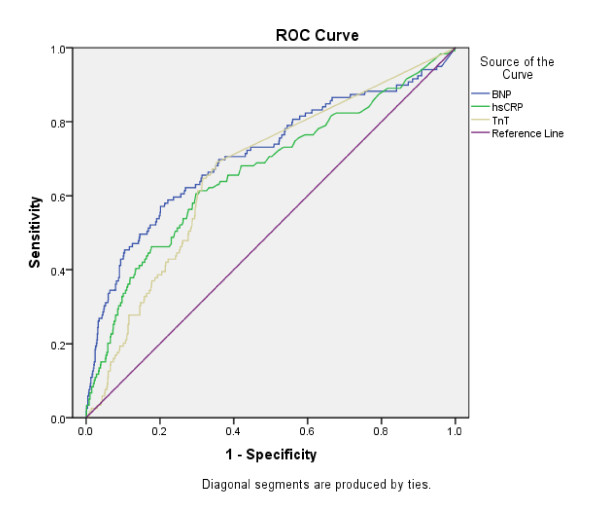
**Receiver operated characteristic curve for BNP, hsCRP and TnT for evaluation of all-cause mortality in total patient population**.

Receiver operated characteristics (ROC) curves for BNP, hsCRP and TnT are shown in Figure 3. The area under the ROC for BNP, hsCRP and TNT was 0.711 (p = 0.000), 0.666 (p = 0.000) and 0.666 (p = 0.000), respectively.

In a stepwise multivariate Cox regression model, BNP was found to be a prognostic indicator of 2 year total mortality in the total patient population. The HR for BNP in Q4 was 2.32 (95% CI, 1.24-4.35) as compared to Q1, which was statistically highly significant, p = 0.009.

In the multivariate Cox regression model hsCRP levels also showed a significant relation to prognosis, with a HR of 1.97 (95% CI, 1.17-3.32), p = 0.011.

#### Troponin-T Positive Patients

In the 388 patients admitted with TnT release we found that 82 patients (21.1%) had died during the 2-year follow-up. The BNP levels were significantly higher among patients dying than in the 2-year survivors. In the univariate analysis for BNP the HR was 3.83 (95% CI, 2.05-7.15), p = 0.000, and in a stepwise multivariable Cox regression model, the HR was 2.12 (95% CI, 1.07-4.18), p = 0.031, remaining significant when age, CHF and CHD were added.

The hsCRP levels were also significantly higher among TnT positive patients with fatal events than in survivors. In the univariate analysis the HR was 3.10 (95% CI, 1.67-5.75, p = 0.012, and in the multivariable model the HR for hsCRP was 3.27 (95% CI, 1.71-6.24), p = 0.000, remaining significant when age, sex and CHF were added.

**Figure 4:**
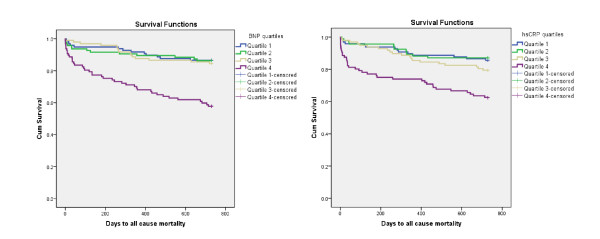
**Kaplan-Meier plots for the cumulative risk of total mortality in the TnT positive patients**.

Kaplan-Meier plots for the cumulative risk of total mortality in the TnT positive patients for BNP and hsCRP quartiles are presented in Figure 4.

#### Troponin-T Negative Patients

**Figure 5:**
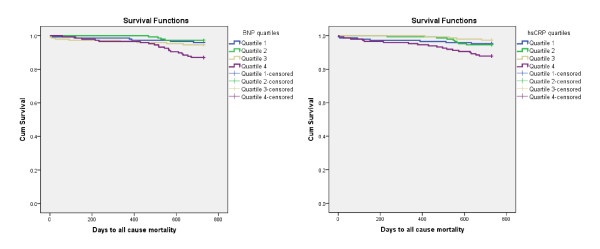
**Kaplan-Meier plots for the cumulative risk of total mortality in the TnT negative patients**.

After a follow-up period of 24 months, 37 patients (6.3%) of 590 with negative TnT results had died. In this group, neither BNP nor hsCRP added any prognostic information. Kaplan-Meier plots for the cumulative risk of total mortality in the TnT negative patients for BNP and hsCRP quartiles are presented in Figure 5.

### Cardiac Death

#### Total Patient Population

After a follow-up period of 24 months, 66 patients (6.9%) had experienced cardiac death.

In the univariate analysis for the total population, the HRs for BNP and hsCRP were 6.97 (95% CI, 2.94-16.54), p = 0.000 and 2.25 (95% CI, 1.19-4.28), p = 0.013, respectively. In a stepwise multivariable Cox regression model, the HR for BNP was 3.34 (95% CI 1.26-8.85), p = 0.015, whereas the HR for hsCRP was not significant, p = 0.21.

#### Troponin-T Positive Patients

The BNP levels in the TnT positive patients were significantly higher among those with cardiac death than in survivors (200.5 (79.5-612.4) versus 73.9 (35.0-159.3) mg/dl [median, 25 and 75% percentiles], p = 0.000).

In this subgroup, the HRs for BNP were 7.07 (95% CI, 2.45-20.46), p = 0.000 and 3.42 (95% CI, 1.13-10.32), p = 0.029, in the univariate and multivariate analyses, respectively. The impact of BNP in Q4 remained significant when age was added to the multivariable model.

In the TnT positive patients the hsCRP levels were also significantly higher among patients with cardiac death than in those that did not have a cardiac death (9.6 (3.0-44.9) versus 4.4 (1.9-12.6) mg/L [median, 25 and 75% percentiles], p = 0.012). Comparing Q4 to Q1 in the multivariate Cox regression model, the HR for hsCRP for cardiac death in the TnT positive patients was 3.05 (95% CI, 1.31-7.11) and reached statistical significance, p = 0.010, but its significance was attenuated (p = 0.33) when adjusted for BNP.

#### Troponin-T Negative Patients

Neither BNP nor hsCRP added any prognostic information in this subpopulation.

## Discussion

The present prospective observational study was carefully designed to meet the requirements for a prognostic evaluation of biomarkers. Contrary to randomized studies, patients are unselected and included on a consecutive basis which offers a great advantage for risk identification.

In this study we have included admission samples of the two biomarkers BNP and hsCRP, to investigate their impact on prognosis in patients with chest pain and suspected ACS, and we have discriminated between patients with and without a release of TNT.

It has been suggested that the combined assessment of natriuretic peptides and CRP may yield incremental prognostic information in the risk stratification of patients with ACS [2,3], and their combined use has been shown to improve long-term risk prediction of mortality in patients with stable coronary heart disease (CHD) [4].

In the stepwise multivariate model applied to the total chest-pain patient material we included the presence and absence of a TnT release and were able to demonstrate a statistical significant prognostic impact of BNP and hsCRP, respectively, on 2 year survival, both for total and cardiac mortality.

In the TnT positive subgroup we found a statistically significant prognostic impact of BNP in the stepwise multivariate Cox regression model on all cause mortality and cardiac mortality, whereas hsCRP was found to be related only to total mortality.

In a univariate discriminant analysis we found that a TnT positive event was correctly classified by BNP or hsCRP in over 60% of cases. The specificity of BNP and hsCRP for predicting all cause mortality of the total population was around 90% for both biomarkers, associated with a sensitivity of 44.5 and 31.9%, respectively.

In the present study, patients in the highest quartile of both BNP and hsCRP were older and a higher proportion had a TnT exceeding 0.01 ng/ml. In the highest quartile of BNP there were also more past smokers and subjects with established CHD and HF, and creatinine was also increased. These differences reflect the increased burden of risk in the upper quartiles of BNP and hsCRP, respectively. Despite some similarities in underlying risk burden, these two predictors are mechanistically different. However, their combination did not strengthen the prognostic utility.

Our results indicate that both BNP and hsCRP are major predictors of outcome in a population in which invasive coronary intervention is less available as compared to wealthier communities, as only 29% of the total population and 38% of the TnT positive population underwent a revascularization procedure during the hospitalization for the index event.

Thirty-one percent of the TnT positive population was classified as STEMIs, and of these patients only 42% were treated with primary PCI. Furthermore, the use of thrombolytic therapy in this region of Argentina is uncommon and was not applied in our patient cohort. The less frequent use of reperfusion treatment in STEMI patients makes this population unique in an epidemiological setting, optimizing an evaluation of prognostic indicators in relation to the natural course of disease.

The results of this observational study is in accordance with previous studies investigating the prognostic impact of BNP [21] and hsCRP [22], and also emphasize the importance of several clinical background factors in this respect [23-25]. After adjusting for clinical covariates, the prognostic utility of BNP as well as hsCRP remained statistically significant with respect to total mortality in both the total population and in the TnT positive patients.

It was shown in the mid 90-ies that elevated troponins are associated with a worsened prognosis in ACS patients [26]. Since then, a considerable number of publications have verified the prognostic significance of this biomarker in this patient setting [27]. Its prognostic utility exceeds that of all other biomarkers, including BNP [8] and hsCRP [14].

Based on previous studies [14,15,22,28] addressing the prognostic utility of hsCRP, this biomarker has been considered for adoption into risk assessment algorithms [1,29]. Recently conducted studies show that the predictivity of hsCRP is attenuated when tested in a multivariable model in the general population [30] and together with natriuretic peptides in patients with known CAD [31-34]. In our study hsCRP seems to be a potential predictor for all cause mortality, also when adjusted for BNP, but does not reflect cardiac mortality in the TnT positive population when introducing BNP into the model.

In contrast to hsCRP, natriuretic peptides integrate specific pathophysiological signals, especially relating to ventricular dysfunction [5,35] and ischemic burden [9]. The main prognostic impact of BNP was found in the TnT positive patients, suggesting a relation to ischemia.

The measurement of prognostic biomarkers has often been performed in randomized interventional trials several days following the onset of symptoms and hospitalization [8,10,29]. In contrast to the majority of previous studies investigating the prognostic impact of various biomarkers, our study had a prospective and observational design, and blood samples were collected directly on admission.

Few studies have examined the predictive value of natriuretic peptides across the spectrum of chest-pain patients with suspected ACS in blood samples obtained directly on admission before introduction of therapy [22]. Therefore, in the present study which is similar to a previous study performed by our group [36], we do not have to consider the potential confounding factors of late inclusions and recently introduced medical treatment. Similar considerations apply to the measurement of hsCRP.

A major strength of this study is the absence of patients lost to follow-up and there were only four patients with no measurements available for hsCRP. Moreover, our study was performed in an inhomogeneous and unselected chest-pain population with suspected ACS which is representative of the one commonly dealt with in the ED.

Our study suggests that BNP and/or hsCRP in addition to the troponins may be supplementary biomarkers in risk stratification. However, their impact in the clinical prognostic assessment of ACS patients depends on the presence of troponin release. By including patients with borderline troponin levels ≤ 0.05 ng/mL among the TnT negative patients and without adjusting for a positive troponin value, the HR of BNP for cardiac death in Q4 as compared to Q1 in the multivariate Cox regression model was 3.58 (95% CI, 1.02-12.60), p = 0.047, in this extended patient category. The same relation was observed for hsCRP; HR of 2.69 (95% CI, 1.22-5.95), p = 0.015, suggesting that the use of BNP or hsCRP may strengthen the clinical prognostic assessment in this subgroup.

### Limitations

The potential limitations of these data merit consideration. The circulating concentrations of BNP and hsCRP prior to hospitalization remain unknown and our analyses are based on a single baseline determination. Although we did not adjust for LVEF, we did adjust for known CHF and CVD, including previous MI, and other clinical risk factors.

## Conclusion

BNP and hsCRP may act as clinically useful prognostic biomarkers when obtained at hospital admission in an unselected chest-pain population with potential ACS, and may improve risk stratification in troponin positive patients. However, these biomarkers failed to identify patients at risk in the troponin negative population.

## Competing interests

The authors declare that they have no competing interests.

## Authors' contributions

RLF: Contributed to study design, data collection, clinical follow-up, interpretation of results and preparation of the manuscript. PAN: Contributed to data collection, clinical follow up, interpretation of results and preparation of the manuscript. STN: Contributed to study design, interpretation of the results and commented on the manuscript. LW: Contributed to study design and commented on the manuscript. TA: Contributed to data collection, clinical follow-up and commented on the manuscript. PG: Contributed to data collection and clinical follow-up. HG: Contributed to assembly of data, interpretation of results and commented on the manuscript. HS: Performed the statistical analysis, contributed to the interpretation of the results and commented on the manuscript. DWTN: Conceived the idea of the study, supervised the study including interpretation of results and preparation of the manuscript.

All authors have read and approved the final manuscript.

## Pre-publication history

The pre-publication history for this paper can be accessed here:

http://www.biomedcentral.com/1471-2261/11/57/prepub
